# Oral minocycline therapy as first-line treatment in patients with Myalgic encephalomyelitis and long COVID: A pilot study

**DOI:** 10.1016/j.ensci.2024.100537

**Published:** 2024-12-02

**Authors:** Kunihisa Miwa

**Affiliations:** Department of Internal Medicine, Miwa Naika Clinic, Toyama, Japan

**Keywords:** Minocycline, Myalgic encephalomyelitis, Chronic fatigue syndrome, Long COVID, Disequilibrium, Post-coronavirus vaccination sequalae

## Abstract

**Background:**

Myalgic encephalomyelitis (ME) is associated with long COVID and also untoward sequelae after anti-coronavirus vaccination. Recently, oral minocycline therapy has been reported to ameliorate symptoms in patients with ME, particularly at the initial stage of the disease.

**Methods:**

Oral minocycline (100 mg × 2 on the first day, followed by 100 mg/day for 41 days) was administered to 55 patients with ME that emerged during the “Corona era,” including 19 patients with long COVID and 5 patients diagnosed with untoward sequalae following coronavirus vaccination.

**Results:**

Acute adverse effects including nausea and/or dizziness caused four (7 %) patients to discontinue treatment in the first few days. Among the other 51 patients who completed therapy, favorable effects were observed, including a decrease in performance status score or index for restricted activities of daily living ≥2 points in 41 (80 %) patients. Disease duration was inversely associated with the favorable therapeutic effects (*p* = 0.02) and the disease duration within 6 months was significantly associated with the favorable therapeutic effects (27/30, 90 %, *p* = 0.02, hazard ratio: 3.6, 95 % confidence interval, 1.2**–**10.6). The favorable effects were observed in 16 (89 %) of 18 patients with long COVID. Significant amelioration of subjective symptoms of fatigue, post-exertional malaise, unrefreshing sleep, brain fog, disequilibrium, orthostatic intolerance, and neuropathic pain were observed.

**Conclusions:**

Oral minocycline was effective at ameliorating symptoms in patients with ME including long COVID and post-coronavirus vaccination sequalae. It represents an effective first-line therapeutic option for these patients in the initial stage.

## Highlights

• The clinical presentation of long COVID resembles that of myalgic encephalomyelitis (ME).
• Minocycline ameliorates neural inflammation.
• Oral minocycline therapy administered as a 6-week regimen was effective in ameliorating symptoms in patients with myalgic encephalomyelitis and long COVID.
• This therapy may be an effective first-line therapeutic option for patients, particularly at the initial stage of disease (<6 months).

## List of Abbreviations

**Table:** 

ME	Myalgic encephalomyelitis
CFS	Chronic fatigue syndrome

## 1 Introduction

Dysfunction of the central nervous system resulting from neuroinflammation with myalgic encephalomyelitis (ME) has been postulated as the cause of chronic fatigue syndrome (CFS) [1,2]. This disease is characterized by severe disabling fatigue, prolonged postexertional malaise, and unrefreshing sleep, leading to a marked reduction in daily living activities and impaired quality of life. It occurs in many young people, mostly women, who lose their ability to work [1,2]. Despite this public health burden, an effective treatment for ME remains to be established.

**Fig. 1:**
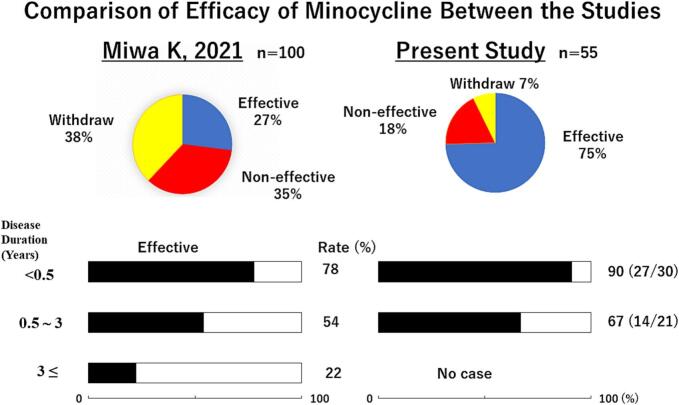
Upper: Note that the rate of favorable effects is much higher and the rate of withdrawal is much lower in the present study compared with that in the Miwa K 2021 study [3]. Lower: The black portion in each bar graph represents the patients with favorable therapeutic effects from oral minocycline among those with a different duration of disease. The rates in the present study and those of Miwa K 2021 [3] were similar for the patients with identical disease duration. See text for details.

Recently, oral minocycline therapy has been reported to exert favorable therapeutic effects in some patients with ME, particularly at the initial stage of the disease; however, many patients discontinued treatment in the first few days because of acute adverse effects such as nausea and/or dizziness (Fig. 1) [3]. Minocycline exerts a variety of biological effects against neural inflammation that are independent of its antimicrobial activity, including anti-inflammatory, immunomodulatory, and neuroprotective effects [4,5].

In recent years, it has been observed that COVID-19 disease may cause persistent signs and symptoms described as post-COVID syndrome or long COVID [6]. There is significant overlap in the onset, symptom profile and progression of long COVID with ME [[7], [8], [9]]. A wide range of infectious agents have been suggested to trigger the development of ME and one such pathogen may be the COVID-19 virus [2].

Recently, I had a valuable experience with a 22-year-old female patient who presented long COVID over a 14-month duration. She completely recovered from ME-like symptoms after treatment with oral minocycline [10]. This case suggests that oral minocycline may be an effective first-line therapy for long COVID.

In the present study, the therapeutic effects of oral administration of minocycline were examined in patients who developed ME during the “Corona era,” including ME as long COVID [9] and also untoward sequalae after receiving a coronavirus vaccine.

## 2 Materials and methods

### 2.1 Study participants

The study population consisted of 55 consecutive patients (18 men and 37 women, mean age: 35 ± 16 years) at the author's clinic between April 2020 and January 2024. The inclusion criteria were as follows: ME diagnosis based on the 2011 International Consensus Criteria [2] with ≥2 months of disease duration prior to the study, an ability to stand and walk, and informed consent to participate. Alternative diagnoses for fatigue and other symptoms were ruled out. The population included 19 (35 %) patients with ME, which developed as long COVID diagnosed ≥3 months after Covid-19 infection based on WHO criteria [7], and 5 (9 %) patients diagnosed with sequalae following coronavirus vaccination. Besides symptoms of long COVID with evidenced Covid-19 infection, fever or onset of infection was reported by 10 (18 %) of the patients.

All the study participants gave written informed consent, and the study was approved by the Ethics Committee of the institute and was conducted under the Declaration of Helsinki. Also, each participant gave written informed consent for possible publication of identifying information/images in an online open-access publication.

### 2.2 Procedures

Oral minocycline was administered on a 6-week schedule (100 mg × 2 on the first day, followed by 100 mg/day for 41 days) [3] to 55 study ME patients.

To evaluate the therapeutic effects, all patients underwent performance status (PS) grading for general condition. Based on subjective symptom severity reported by the patients as described previously, PS was graded on a 10-point scale as below [11] (Table 1).

**Table 1:** Performance status (PS) grading.

PS 0:	The patient can perform usual activities of daily living and social activities without malaise.
PS 1:	The patient often feels fatigue.
PS 2:	The patient often needs to rest because of general malaise or fatigue.
PS 3:	The patient cannot work or perform usual activities for a few days in a month.
PS 4:	The patient cannot work or perform usual activities for a few days in a week.
PS 5:	The patient cannot work or perform usual activities but can perform light work.
PS 6:	The patient needs daily rest but can perform light work on a “good day”.
PS 7:	The patient can take care of himself/herself but cannot perform usual duties.
PS 8:	The patient needs help to take care of himself/herself.
PS 9:	The patient needs to rest the whole day and cannot take care of himself/herself without help.

To evaluate specific symptoms, neurologic examinations [[11], [12], [13]], conventional active 10 min standing test [11], and digital palpation for 18 specified tender points proposed by the American College of Rheumatology in 1990 [14] were conducted. In addition, a subjective evaluation for various symptoms, including fatigue, post-exertional malaise, unrefreshing sleep, and cognitive dysfunction or “Brain fog” were performed. Symptom severity was measured using a Likert scale (no symptom, very mild, mild, moderate, severe, very severe). After verification by the only doctor (author), the symptom was counted as positive when it was moderate, severe or very severe and as negative when it was no symptom, very mild or mild. All tests were conducted before initiating minocycline therapy and within 2 months after therapy. Ongoing medications including nutritional supplements and multi-enzyme tablets were continued throughout the study.

### 2.3 Statistical analysis

Continuous variables are presented as median with interquartile range, although age is presented as mean ± standard deviation. Proportional data were analyzed using the Fisher's exact test with Yates' correction. Wilcoxon signed-rank test was used to compare both median PS scores between the study patients before and after treatment with oral minocycline. Independent factors associated with the favorable therapeutic effects were evaluated using a Cox regression analysis. Statistical significance was set at *p* < 0.05.

## 3 Results

Among the 55 study patients who began oral minocycline, four (7 %) including one with long COVID experienced adverse effects during the first three days and stopped taking the drug, primarily because of severe nausea and/or dizziness (Fig. 1). The remaining other 51 (93 %) patients including 18 with long COVID completed the 6-week course of treatment.

### 3.1 Performance status scoring

Among the 51 patients who completed treatment, the PS score decreased by at least two in 41 patients (80 %), whereas it was essentially unchanged in the remaining 10 patients (20 %). As shown in Fig. 1, favorable therapeutic effects were prevalently observed in patients with a shorter (<6 months) disease duration (27/30, 90 %). A Cox regression analysis revealed that disease duration was inversely associated with the favorable therapeutic effects (*p* = 0.02) and the disease duration within 6 months was significantly associated with the favorable therapeutic effects (p = 0.02, hazard ratio: 3.6, 95 % confidence interval, 1.2**–**10.6) while age, sex, long COVID, and disequilibrium were not.

### 3.2 Favorable effects in patients with long COVID

A markedly high rate of the favorable effects was observed in 16 (89 %) of 18 patients with long COVID who completed treatment (Table 2). The remaining one patient stopped taking the drug on the second day due to severe nausea and dizziness. Totally the rate of the favorable effects was 84 % (16/19).

**Table 2:** Effects of oral minocycline on various symptoms in patients with ME and long COVID.

Patient	Age/Sex	Disease	Performance	Fatigue	Post-exertional	Unrefreshing	Brain fog	Orthostatic	Disequilibrium	Tender
#		Duration (M)	status score		malaise	sleep		intolerance		points
1	22/F	13	6 → 1	+ → -	+ → -	+ → -	- → -	- → -	- → -	12 → 0
2	33/F	3	7 → 4	+ → +	+ → +	+ → +	+ → +	+ → -	+ → -	0 → 0
3	26/F	3	6 → 3	+ → +	+ → +	+ → +	+ → -	- → -	- → -	6 → 0
4	51/M	3	7 → 1	+ → -	+ → -	+ → -	+ → -	- → -	- → -	0 → 0
5	30/M	3	4 → 5	+ → +	+ → +	+ → +	+ → +	- → +	+ → +	8 → 4
6	15/F	3	3 → 1	+ → -	+ → -	+ → -	- → -	- → -	- → -	0 → 0
7	37/M	3	4 → 1	+ → -	+ → -	+ → -	- → -	- → -	- → -	2 → 0
8	36/F	6	6 → 1	+ → -	+ → -	+ → -	+ → -	- → -	+ → -	12 → 0
9	26/F	9	7 → 4	+ → -	+ → -	+ → -	+ → -	+ → -	+ → -	18 → 0
10	59/M	2	4 → 1	+ → -	+ → -	+ → -	- → -	+ → -	+ → -	0 → 0
11	63/F	2	3 → 0	+ → -	+ → -	- → -	- → -	- → -	- → -	0 → 0
12	77/F	12	4 → 2	+ → -	+ → -	+ → -	- → -	- → -	+ → +	0 → 0
13	15/M	2	3 → 1	+ → -	+ → -	+ → -	- → -	- → -	- → -	0 → 0
14	56/M	11	6 → 3	+ → -	+ → -	+ → -	- → -	- → -	+ → -	0 → 0
15	37/F	9	6 → 1	+ → -	+ → -	- → -	- → -	- → -	- → -	0 → 0
16	24/M	4	6 → 3	+ → -	+ → -	+ → -	- → -	- → -	- → -	0 → 0
17	15/M	11	3 → 1	+ → -	+ → -	+ → -	+ → -	- → -	+ → -	0 → 0
18	23/F	17	4 → 3	+ → +	+ → +	+ → +	+ → +	- → -	- → -	12 → 0
Total	36 8M	6.4	median							≥8
	±18 10F	±4.7	5 → 1*	18+ → 4 + *	18+ → 4 + *	16+ → 4 + *	8+ → 3+	3+ → 1+	8+ → 2+	5 → 0**

*p < 0.01 **p < 0.05 vs. before treatment.

Dysosmia and/or dysgeusia were present in patients #9 and 15 before treatment and ameliorated after therapy.

ME: Myalgic encephalomyelitis; Tender points by ACR1990 [14]; See text for details.

### 3.3 Disequilibrium

Before therapy, 21 patients had disequilibrium with an unstable tandem gait [[11], [12], [13]]. After treatment, 16 (76 %) exhibited resolution of their disequilibrium (Table 3). Typical cases with recovered disequilibrium after treatment with oral minocycline are presented (see Supplemental Material). (See Table 4.)

**Table 3:** Comparison of the performance status scores and the prevalence of various symptoms between the study 47 patients with ME before and after the treatment with minocycline.

	Before minocycline	After minocycline	*P* value
Performance status score	3–8	0–7	
Median score (IQR)	5 (4–7)	3 (1–5)	<0.01
Fatigue	46 (98 %)	14 (30 %)	<0.01
Postexertional malaise	45 (96 %)	14 (30 %)	<0.01
Unrefreshing sleep	41 (87 %)	10 (21 %)	<0.01
Brain fog	34 (72 %)	9 (19 %)	<0.01
Orthostatic intolerance	8 (17 %)	1 (2 %)	0.03
Disequilibrium	19 (40 %)	5 (11 %)	<0.01
Neuropathic pain	12 (25 %)	4 (9 %)	0.052
Dysosmia/Dysgeusia	4 (9 %)	1 (2 %)	0.21

ME: myalgic encephalomyelitis; IQR: interquartile range; Orthostatic intolerance: inability to keep standing for 10 min; Disequilibrium: unstable tandem gait; Neuropathic pain: tender points ≥ 8.

**Table 4:** Comparison of the performance status scores and the prevalence of various symptoms between the study 18 ME patients with long COVID before and after the treatment with minocycline.

	Before minocycline	After minocycline	*P* value
Performance status score	3–7	0–5	
Median score (IQR)	5 (4–6)	1 (1–3)	<0.01
Fatigue	18 (100 %)	3 (20 %)	<0.01
Postexertional malaise	18 (100 %)	3 (20 %)	<0.01
Unrefreshing sleep	16 (89 %)	3 (20 %)	<0.01
Brain fog	8 (41 %)	2 (13 %)	0.15
Orthostatic intolerance	3 (17 %)	1 (7 %)	0.35
Disequilibrium	8 (44 %)	2 (13 %)	0.06
Neuropathic pain	5 (28 %)	0 (0 %)	0.045
Dysosmia/Dysgeusia	2 (11 %)	0 (0 %)	0.24

ME: myalgic encephalomyelitis; IQR: interquartile range); Orthostatic intolerance: inability to keep standing for 10 min; Disequilibrium: unstable tandem gait; Neuropathic pain: tender points ≥ 8.

### 3.4 Active 10 min standing test

Before therapy, 8 patients were unable to complete the 10 min standing test with orthostatic intolerance. After therapy, 7 (88 %) of 8 completed the test with recovered orthostatic intolerance (Table 3).

### 3.5 Neuropathic pain or fibromyalgia

Before therapy, 13 patients were diagnosed with neuropathic pain (tender points ≥ 8). In 9 (69 %) patients, the number of tender points decreased markedly (≥4) after treatment (Table 3).

### 3.6 Comparison of PS scores and prevalence of various specific symptoms before and after treatment

**Fig. 2:**
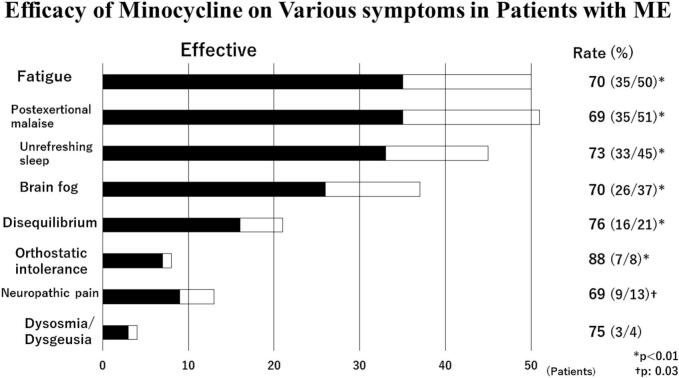
The rate of favorable therapeutic effects following oral minocycline treatment on various symptoms in the study patients. The black portion in each bar graph shows the patients with favorable therapeutic effects. *p < 0.01 †*p* = 0.03.

Comparison of PS scores and prevalence of various symptoms in study 51 ME patients who completed treatment before and after treatment are shown in Table 3. The median PS score and also prevalence of various symptoms and signs including fatigue, post-exertional malaise, unrefreshing sleep, brain fog, orthostatic intolerance, disequilibrium, and neuropathic pain were significantly lower in the patients after treatment as compared with before (Fig. 2).

Comparison of PS scores and prevalence of various symptoms in 18 ME patients with long COVID who completed treatment before and after treatment are shown in Tables 2 and 4. The median PS score and prevalence of various symptoms and signs including fatigue, post-exertional malaise, unrefreshing sleep, and neuropathic pain were significantly lower in the patients after treatment as compared with before. Fatigue (80 %), post-exertional malaise (80 %), unrefreshing sleep (81 %), and neuropathic pain (100 %) were all significantly ameliorated following treatment in the patients.

### 3.7 Comparison between the results of the present study and a previous report

Figure 1 shows the comparative rate with favorable effects by PS grading according to disease duration in the present study and a previous study [3] with ME patients before the “Corona era.” Although no patients with a disease duration ≥3 years were included in the present study, the rate of favorable effects appeared to be disease duration-dependent. The most favorable effect rate was observed among patients with a short disease duration (<6 months) in the present study, which was similar to the previous study.

## 4 Discussion

Several pathobiological mechanisms have been proposed for ME/CFS, including redox imbalance, immune abnormalities, central nervous system inflammation, energy production/transport impairments, mitochondrial alterations, as well as endothelial and vascular disturbances [2,[15], [16], [17]]. Recently, these mechanisms have been unified into a common pathophysiological hypothesis centered on the pathologically reactive neuroglia (mainly microglia and astrocytes) in stress-responsive brain regions, such as the hypothalamic nuclei and other parts of the limbic system. This hypothesis can efficiently explain not only the onset of ME/CFS symptoms but also the hallmark features of the disorder, such as post-exertional malaise, and its progression over time [18]. On the other hand, acute systemic and central nervous system inflammation, reactive pro-inflammatory neuroglia, oxidative stress and abnormally elevated levels of pro-inflammatory cytokines have also been reported in patients with long COVID [[19], [20], [21]]. A pathobiological model for long COVID and ME/CFS based on their common inflammatory and dysfunctional glial reactivity has been proposed [22].

The similar symptoms and pathology of ME/CFS and long COVID suggest that these disorders represent just two examples of a broader illness, because they occur through a carefully orchestrated, stereotyped, multi-system response to infection and injury [9]. Indeed, many patients with severe long Covid are diagnosed as ME/CFS based on various criteria [1,2,7,9,23,24]. Approximately one-third of the patients in the present study who developed ME that was diagnosed according to ICC during the “Corona era” also had long COVID.

In the present study, favorable therapeutic effects of oral minocycline were observed in patients who developed ME during the “Corona era.” The rate of favorable effects observed for varying disease duration were similar between the present study and a previous one [3]. A shorter disease duration was prevalent in patients from the present study, in which greater total therapeutic effects were observed. Also, the withdrawal of oral minocycline therapy was much less prevalent in the present study compared with the previous one (Fig. 1). Longer disease duration may change functional neuroinflammation into stable organic neural damage, which is resistant to therapy. Also, longer disease duration of ME (>3 years) was associated with worse drug sensitivity and untoward effects, which were probably related to affinity maturation that developed over a long disease period. Recently, the treatment with repetitive transcranial magnetic stimulation, a newly developed neuromodulatory procedure, has been reported to be effective in alleviating various symptoms, especially orthostatic intolerance and disequilibrium, and in improving activities of daily living in patients with ME [25].

The present study is the first systematic report of the use of oral minocycline therapy for patients with ME and long COVID. Minocycline therapy improved the daily functional capacity or activity of daily living; reduced the symptoms of orthostatic intolerance, which is a primary determinant of daily living activities [26], and disequilibrium, which was recently suggested as an important cause of orthostatic intolerance [[11], [12], [13]]. It also ameliorated myalgic symptoms.

Although dysfunction of the central nervous system with reactive pro-inflammatory microglia may be promising targets in the treatment of ME patients, further understanding of the molecular mechanisms involved in the effects of minocycline on neuroinflammation is needed to achieve its full therapeutic potential.

## 5 Limitations

The present study has some limitations. First, the design was not a double-blind randomized controlled trial; therefore, possible placebo effects could not be excluded. Second, many patients were included with a short disease duration (<6 months) and no systematic reports on the natural course of disease within the first 6 months were available. Therefore, it cannot be excluded that accentuated improvement of some symptoms may include, in part, the natural course of disease during the early stage. Nevertheless, a markedly high rate of the favorable effects suggests that oral minocycline therapy represents an effective first-line therapeutic option for these patients, although a large scale of trial is needed to justify the therapy as a first-line option.

## 6 Conclusions

Oral minocycline therapy administered as a 6-week regimen was effective at ameliorating symptoms in most patients with ME and long COVID. It represents an effective first-line therapeutic option for these patients, especially at the initial stage of disease (<6 months).

The following are the supplementary data related to this article.

Supplementary Video 1 Supplementary Video 1

Supplementary Video 2 Supplementary Video 2

Supplementary Video 3 Supplementary Video 3

Supplementary Video 4 Supplementary Video 4

Supplementary material 1 Supplementary material 1

## Ethics approval and consent to participants

This study was approved by the institutional ethics committee of Miwa Naika Clinic (approval number: 2020–001).

## Consent for publication

Written consent to publish was obtained from the patients.

## Availability of data

Data that support the findings presented intis study are available from the corresponding author upon reasonable request.

## Funding

This research did not receive any specific grant from funding agencies in the public, commercial, or not-for-profit sectors.

## CRediT authorship contribution statement

**Kunihisa Miwa:** Writing – review & editing, Writing – original draft, Visualization, Validation, Supervision, Software, Resources, Project administration, Methodology, Investigation, Funding acquisition, Formal analysis, Data curation, Conceptualization.

## Declaration of competing interest

None.

## References

[1] Fukuda K., Straus S.E., Hickie I. (1994). The chronic fatigue syndrome: a comprehensive approach to its definition and study. Ann. Intern. Med..

[2] Carruthers B.M., van de Sande M.I., DeMeirleir K.L. (2011). Myalgic encephalomyelitis: international consensus criteria. J. Intern. Med..

[3] Miwa K. (2021). Oral minocycline therapy improves symptoms of myalgic encephalomyelitis, especially in the initial disease stage. Intern. Med..

[4] Garrido-Mesa N., Zarzuelo A., Gálvez J. (2013). Minocycline: far beyond an antibiotic. Br. J. Pharmacol..

[5] Kataoka Y., Yamato M., Miyashige Y. (2013). Neuroinflammation in animal models of fatigue. Adv. Neuroimm. Biol..

[6] Campos M.C., Nery T., Starke A.C. (2022). Post-viral fatigue in COVID-19: a review of symptom assessment methods, mental, cognitive, and physical impairment. Neurosci. Biobehav. Rev..

[7] Soriano J.B., Murthy S., Marshall J.C. (2021). WHO clinical case definition working group. A clinical case definition of post-COVID-19 condition by a Delphi consensus. Lancet Infect. Dis..

[8] Sukocheva O.A., Maksoud R., Beeraka N.M. (2022). Analysis of post COVID-19 condition and its overlap with myalgic encephalomyelitis/chronic fatigue syndrome. J. Adv. Res..

[9] Komaroff A.L., Lipkin W.I. (2023). ME/CFS and long COVID share similar symptoms and biological abnormalities: roadmap to the literature. Front. Med..

[10] Miwa K. (2022). Oral minocycline challenge as a potential first-line therapy for myalgic encephalomyelitis and long Covid-19 syndrome. Ann. Clin. Med. Case Rep.

[11] Miwa K., Inoue Y. (2018). The etiologic relation between disequilibrium and orthostatic intolerance in patients with myalgic encephalomyelitis (chronic fatigue syndrome). J. Cardiol..

[12] Miwa K., Inoue Y. (2017). Truncal ataxia or disequilibrium is unrecognised cause of orthostatic intolerance in patients with myalgic encephalomyelitis. Int. J. Clin. Pract..

[13] Miwa K., Inoue Y. (2020). Paradigm shift to disequilibrium in the genesis of orthostatic intolerance in patients with myalgic encephalomyelitis and chronic fatigue syndrome. Int. J. Cardiol. Hypertens.

[14] Wolfe F., Smythe H.A., Yunus M.B. (1990). The American College of Rheumatology 1990 criteria for the classification of fibromyalgia. Arthritis Rheum..

[15] Holden S., Maksoud R., Eaton-Fitch N. (2020). A systematic review of mitochondrial abnormalities in myalgic encephalomyelitis/chronic fatigue syndrome/systemic exertion intolerance disease. J. Transl. Med..

[16] Mackay A., Tate W.P. (2018). A compromised paraventricular nucleus within a dysfunctional hypothalamus: a novel neuroinflammatory paradigm for ME/CFS. Int. J. Immunopathol. Pharmacol..

[17] Nelson T., Zhang L.-X., Guo H. (2021). Brainstem abnormalities in myalgic encephalomyelitis/chronic fatigue syndrome: a scoping review and evaluation of magnetic resonance imaging findings. Front. Neurol..

[18] Renz-Polster H., Tremblay M.-E., Bienzle D., Fischer J.E. (2022). The pathobiology of myalgic encephalomyelitis/chronic fatigue syndrome: the case for neuroglial failure. Front. Cell. Neurosci..

[19] Meinhardt J., Radke J., Dittmayer C. (2021). Olfactory transmucosal SARS-CoV-2 invasion as a port of central nervous system entry in individuals with COVID-19. Nat. Neurosci..

[20] Poloni T.E., Medici V., Moretti M. (2021). COVID-19–related neuropathology and microglial activation in elderly with and without dementia. Brain Pathol..

[21] Schwabenland M., Sali’e H., Tanevski J. (2021). Deep spatial profiling of human COVID-19 brains reveals neuroinflammation with distinct microanatomical microglia-T-cell interactions. Immunity.

[22] Chaves-Filho A.M., Braniff O., Angelova A. (2023). Chronic inflammation, neuroglial dysfunction, and plasmalogen deficiency as a new pathobiological hypothesis addressing the overlap between post-COVID-19 symptoms and myalgic encephalomyelitis/chronic fatigue syndrome. Brain Res. Bull..

[23] IOM (Institute of Medicine) (2015).

[24] Wong T., Weitzler D. (2021). Long COVID and myalgic encephalomyelitis/chronic fatigue syndrome (ME/CFS) — a systemic review and comparison of clinical presentation and symptomatology. Medicina (Kaunas).

[25] Miwa K., Inoue Y. (2023). Repetitive transcranial magnetic stimulation ameliorates symptoms in patients with myalgic encephalomyelitis (chronic fatigue syndrome). IBRO Neurosci. Rep..

[26] Costigan A., Elliott C., McDonaldo C. (2010). Orthostatic symptoms predict functional capacity in chronic fatigue syndrome: implications for management. Q. J. M..

