# Supplementary material for: Oral minocycline therapy as first-line treatment in patients with Myalgic encephalomyelitis and long COVID: A pilot study
Source: eNeurologicalSci. 2024 Dec 2;38:100537. doi: 10.1016/j.ensci.2024.100537 (PMC11665601; doi:10.1016/j.ensci.2024.100537)
Supplement: Supplementary material 1 [file mmc5.docx]

Supplementary files

A 33-year-old female patient with ME and long COVID (patient #2 in Table 2). Disequilibrium with unstable one-foot standing and tandem gait (File 1) completely recovered (File 2) after treatment with oral minocycline.

A 36-year-old female patient with ME and long COVID (patient #8 in Table 2). Disequilibrium with unstable tandem gait (File 3) completely recovered (File 4) after treatment with oral minocycline.
